# Simulation of the impact of people mobility, vaccination rate, and virus variants on the evolution of Covid-19 outbreak in Italy

**DOI:** 10.1038/s41598-021-02546-y

**Published:** 2021-12-01

**Authors:** Corrado Spinella, Antonio Massimiliano Mio

**Affiliations:** 1 Dipartimento di Scienze Fisiche e Tecnologie per la Materia, Consiglio Nazionale delle Ricerche, Piazzale Aldo Moro 7, 00185 Rome, Italy; 2 Institute for Microelectronics and Microsystems (IMM), Consiglio Nazionale delle Ricerche (CNR), VIII Strada 5, I-95121 Catania, Italy

**Keywords:** Scientific data, Biological physics

## Abstract

We have further extended our compartmental model describing the spread of the infection in Italy. As in our previous work, the model assumes that the time evolution of the observable quantities (number of people still positive to the infection, hospitalized and fatalities cases, healed people, and total number of people that has contracted the infection) depends on average parameters, namely people diffusion coefficient, infection cross-section, and population density. The model provides information on the tight relationship between the variation of the reported infection cases and a well-defined observable physical quantity: the average number of people that lie within the daily displacement area of any single person. With respect to our previous paper, we have extended the analyses to several regions in Italy, characterized by different levels of restrictions and we have correlated them to the diffusion coefficient. Furthermore, the model now includes self-consistent evaluation of the reproduction index, effect of immunization due to vaccination, and potential impact of virus variants on the dynamical evolution of the outbreak. The model fits the epidemic data in Italy, and allows us to strictly relate the time evolution of the number of hospitalized cases and fatalities to the change of people mobility, vaccination rate, and appearance of an initial concentration of people positives for new variants of the virus.

## Introduction

The new coronavirus severe acute respiratory syndrome coronavirus 2 (SARS-CoV-2), initially started in the city of Wuhan, China^1–4^, has transformed into a pandemic that has affected a large number of countries around the world^5–7^. Several studies have been aimed to pointing out the relations between the manifestation of Covid-19^8^ and several comorbidities which may also contribute to serious consequences of Covid-19 pandemic^9,10^, including Obstructive Sleep Apnea (OSA). National governments played a relevant role in providing information about the outbreak, to explain the restrictions and provide basic hygienic rules. Information was spread out by conventional and social media. A primary role in constraining the outbreak was played by Health Care Workers (HCWs). Several countries have evaluated HCWs about Covid-19, gaining important results in terms of the importance of education, work environment and equipment^11–13^. In parallel, models are extremely useful to identify physical key parameters influencing the spread of infection and thus taking appropriate measures to limit serious consequences of the influenza/SARS pandemics^3,4,7,14–30^.

In this work we present a further extension of our theoretical description, describing the spread of the infection in Italy^14^. As in our previous work, the description is based on a compartmental model that allows us to follow the time evolution of the observable quantities characterizing the virus outbreak: people tested positive for the virus, people tested as healed (i.e. negative, after a period from the infection, to the test for the virus), hospitalized people, fatalities, and total number of those who has been infected.

Compartmental models, and in particular SIR (Susceptible-Infectious-Recovered) type models and their derivations provide powerful tools to describe and control infection disease dynamics and they have been widely used to describe COVID-19 outbreak, also using machine learning oriented parameter optimization^21–26^.

As in the present work, several models in literature are devoted to implement different compartments (e.g. positives, healed, hospitalized, deaths,…) in their formal description in order to take into account all the parts acting in the disease spreading. To assess different temporal behaviour, such as the level of restrictions, SIR-based models can exploit time-varying parameters^27,28^.

As a new key point, the present time-varying model, since its previous version^14^, assumes that the spreading of viral infection can be described by a simple diffusion process, controlled mainly by a diffusion coefficient that changes in time accordingly to the people mobility restrictions adopted in the course of the outbreak and it explicitly introduces this quantity in the theoretical equations. Correlations between human mobility and the disease spreading have been observed since the beginning of the Covid-19 outbreak^29^. However, modelling the disease evolution with mobility data, from external database, is often difficult due to the lack of public information or because it requires the introduction of several extra-parameters and assumptions^30^. In the present work, we report on the tight correlation between people mobility and the Covid-19 disease spreading. In particular, we demonstrate that it is possible to extract the mobility by examining only the time evolution of hospitalized and fatalities cases. The model has been applied to the data of the outbreak in Italy as a whole and in three regions (Lombardia, Sicily, Lazio). As a step forward with respect to our previous paper, the effect of vaccination is now included in the model, in order to analyze the best condition of easing the adopted mobility restrictions as a function of the implemented daily vaccination rate.

Most recently, an increasing concern regards the appearance of virus variants, characterized by higher level of transmissibility or symptom severity. The model describes these effects and their impact on the risk of triggering new epidemic waves. Our approach allows to get a fast feedback of the adopted mobility restrictions on the evolution scenarios of the outbreak, based on a fit to the experimental available data.

In Italy, as of January the 31st, 2021, a total of 2,553,032 cases of coronavirus disease 2019 (COVID-19) and 88,516 deaths have been confirmed. For the description of the Covid19 diseases, we use the official data daily diffused by the Italian Civil Protection Department (Dipartimento della Protezione Civile)^31^^.^

This paper has been first submitted on February the 4th, 2021. The epidemiological data considered in this work cover the time span from February the 24th, 2020 to January the 31st, 2021. For the hospitalized people and fatalities, the data were acquired only processing only molecular swabs, while detection of positive cases in general was performed by means of molecular and rapid antigen-testing swabs^31^. Vaccination data were obtained from the dataset of the Italian Health Ministry (Ministero della Salute)^32^.

Data and code are available on our GitHub repository^33^. For each figure, the experimental dataset is indicated in the corresponding folder ‘./figNNN’, where NNN is the figure number.

**Figure 1:**
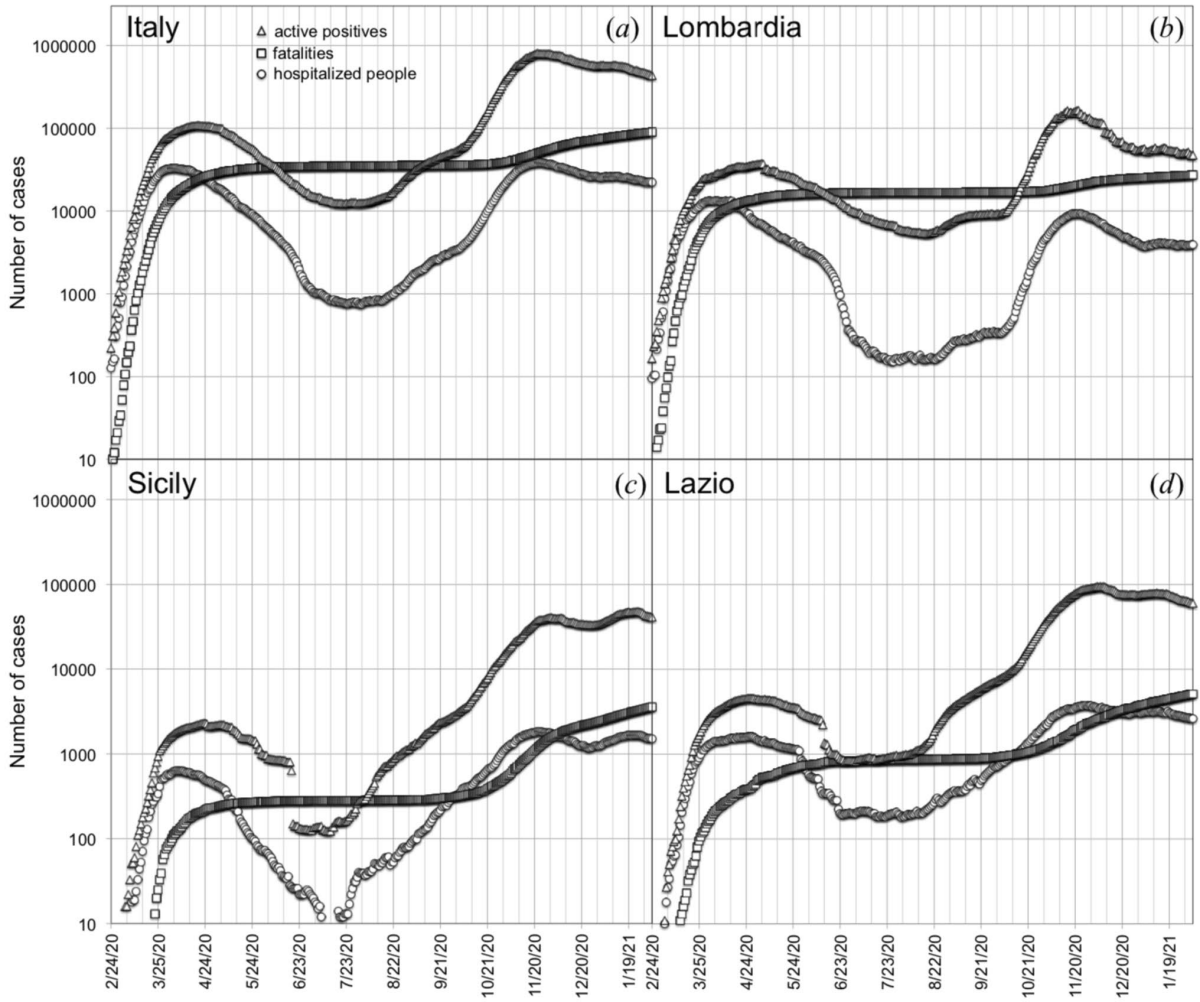
Experimental data of hospitalized people (open circles), fatalities (open squares), people tested positive for the viral infection (open triangles), since the beginning of the Covid-19 outbreak in Italy (**a**), in Lombardia (**b**), in Sicily (**c**), and in Lazio (**d**), respectively.

Epidemiological experimental data (active positives, hospitalized people, fatalities) are shown in Fig. 1, in semi-logarithmic plots as a function of time. The data refer to the numbers of hospitalized people (open circles), people tested positive for the virus (open triangles), and fatalities (open squares) of the Covid-19 outbreak in Italy as a whole (Fig. 1a), and in three different National Italian Regions: Lombardia (Fig. 1b), Sicily (Fig. 1c), and Lazio (Fig. 1d), since February 2020, the 24th. After the initial sudden increase of the numbers, Italy implemented measures aimed to limit people mobility from March 2020 the 9th to June 2020 the 14th. The consequence of such restrictions was a significant slowdown of the outbreak diffusion, following by a decrease in the number of positive and hospitalized cases, extended until end of July 2020. Easing of mobility restrictions induced a new increase of cases, by triggering the second wave of the outbreak lasting until nowadays.

Starting from these considerations, the aims of this work are the following ones:

1. To provide a model able to predict the evolution of the Covid-19 outbreak, especially in Italy, indicating a good forecast as a function of the level of the restrictions;
2. To suggest the best restriction strategies, as a trade-off between restraining the outbreak and facilitating social activities and economics;
3. To include and predict the influence of the vaccination on the previous points.
4. To simulate the impact of the variants in terms of disease spreading increase and of hospitalized persons and the relative incidence in the positive population;
5. To detect the onset of a new variant.

## Methods

The model proposed to describe time evolution of the total number of infected people, positive cases, healed people, deaths, and hospitalized people, during the Covid-19 outbreak is based on a mean-field approximation. This consists in the assumption that the probability for an individual to contract the infection is proportional to the concentration $p$ of positive circulating cases, to a diffusion coefficient $D$, equal to the surface area covered on average by each person in a day, and to an infection cross-section $\sigma$ related to the probability of a single infection event ($\sigma = \pi R^{2}$, $R$ being the average distance within which a healthy person can be infected by a positive one). This cross-section is a quantity specifically dependent on the virus infectiousness. We assume it is constant everywhere all over the examined geographic area and can change only in the presence of virus variants. In particular, the dimensionless probability η of a single infection event is related to the infection cross-section by the relationship $\eta = \rho_{0} \sigma$, where $\rho_{0}$ is the density of inhabitants. Under these hypotheses, at any instant $t$, the increase $dp$ of people positive for viral infection in the time interval $dt$ can be expressed as:

1 $$\frac{dp}{{dt}} = \rho_{0} D\sigma \left( {\rho_{0} - c} \right)p - \frac{dg}{{dt}} - \frac{dm}{{dt}}$$

$g$ is the concentration of healed people (infected people who are tested negative for the virus after a certain time interval from the infection), $m$ is the concentration of fatalities, and $c$ is the total concentration of those who have contracted the virus at the time $t$.

A fraction $f$ of the new positive cases requires hospital care and, consequently, the concentration $r$ of hospitalized people will change, in the time interval $dt$, by a quantity $dr$ given by:

2 $$\frac{dr}{{dt}} = f\rho_{0} D\sigma \left( {\rho_{0} - c} \right)p - \left( {\frac{q}{{\tau_{1} }} + \frac{1 - q}{{\tau_{2} }}} \right)r$$

where we further consider that $r$ diminishes, in the same time interval $dt$, because a fraction $q$ of hospitalized people dies in a characteristic time $\tau_{1}$, whilst the complementary fraction $\left( {1 - q} \right)$ heals in a characteristic time $\tau_{2}$. As a consequence, the concentration $m$ of fatalities will vary with time according to the following equation:

3 $$\frac{dm}{{dt}} = \frac{q}{{\tau_{1} }}r$$

While the fraction $f$ of positive people is hospitalized, the fraction $\left( {1 - f} \right)$ does not exhibit serious symptoms until complete healing. The relative concentration $s$, of people not exhibiting serious symptoms (i.e. not requiring hospital care), will vary with time according to the following relationship:

4 $$\frac{ds}{{dt}} = \left( {1 - f} \right)\rho_{0} D\sigma \left( {\rho_{0} - c} \right)p - \frac{s}{{\tau_{3} }}$$

$\tau_{3}$ being the characteristic time toward healing for these individuals. As reported in Ref.^34^, this characteristic time is typically larger than $\tau_{2}$, i.e. the one used for describing time dependent healing of the most severe hospitalized cases (Eq. 2). As a consequence, the time dependence of the concentration $g$ of healed people changes with time according to:

5 $$\frac{dg}{{dt}} = \frac{s}{{\tau_{3} }} + \frac{{\left( {1 - q} \right)r}}{{\tau_{2} }}$$

Finally, the total concentration $c$ of those who have contracted the infection will vary on time according to the following relationship:

6 $$\frac{dc}{{dt}} = \rho_{0} D\sigma \left( {\rho_{0} - c} \right)p$$

Our description is based on the assumption that the dynamics of all the observable variables, $p$, $r$, $m$,$g$, $c$, can be described in terms of the time dependence of the diffusion coefficient $D\left( t \right)$, while keeping constant the infection cross-section to the value of $= 3.14$ m^2^ (corresponding to $R = 1$ m).

## Results and discussion

### Modelling the epidemic evolution in Italy before 2020 holiday season

**Figure 2:**
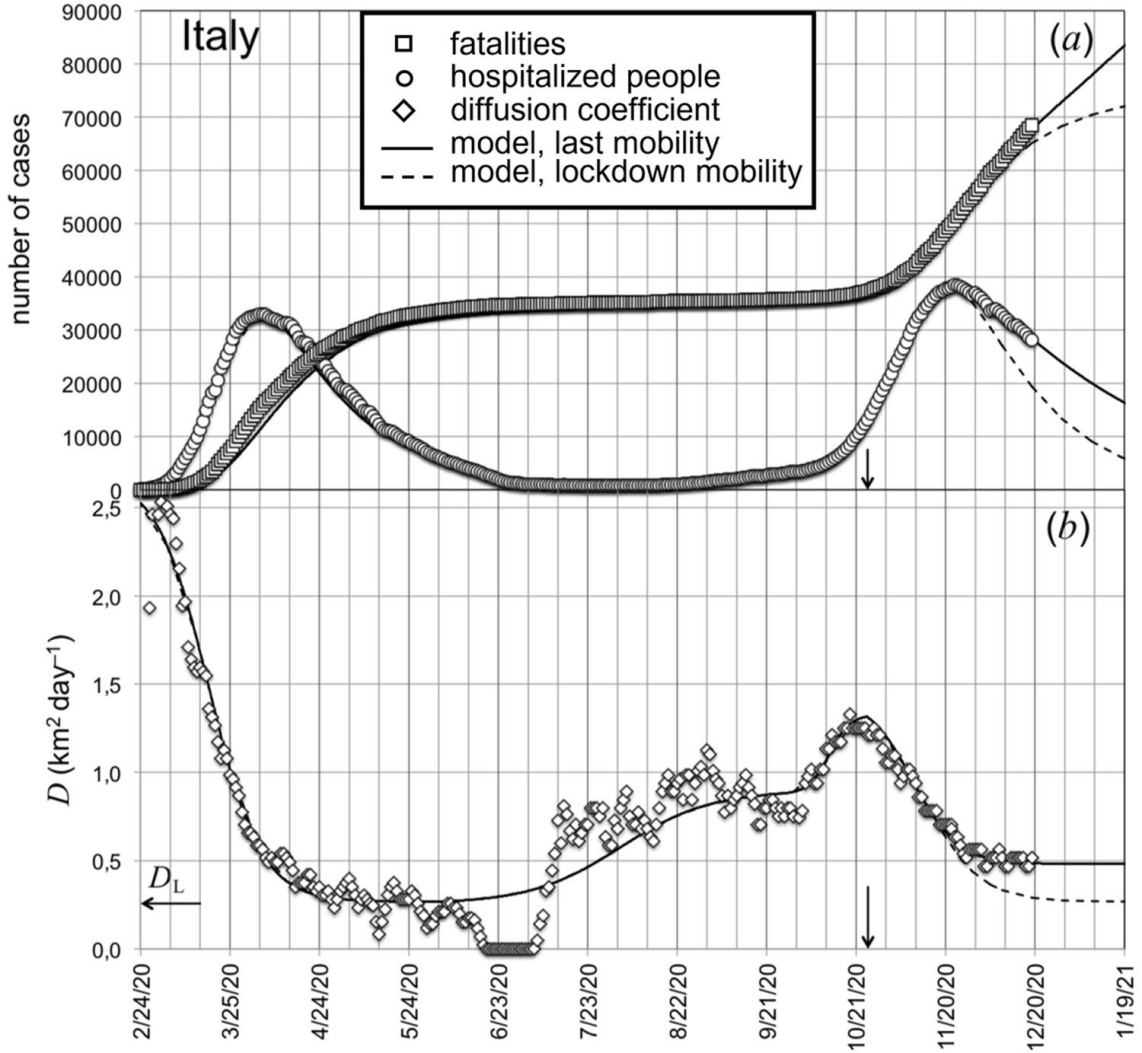
(**a**) Evolution of the data of hospitalized people (open circles) and fatalities (open squares) in Italy during the Covid-19 outbreak until December the 20th, 2020. (**b**) Corresponding values of the diffusion coefficient (open lozenges) extracted from the data of hospitalized cases. Continuous line in (**b**) is fit to the *D* values by using a set of logistic functions (Eq. 7). This functional form is used to model hospitalized cases and fatalities represented by the continuous lines plotted in (**a**). Dashed lines represent the model simulation corresponding to a decrease of the diffusion coefficient, after October the 20th, to the level, *D*_L_, reached during the global spring lockdown.

The values of the diffusion coefficient *D*, from February the 24th, 2020 until December the 20th, 2020 (i.e. a few days before holiday season in Italy), are plotted in Fig. 2b (open lozenges). These values were extracted from the data of the hospitalized cases [open circles in Fig. 2a] by adopting the analytical procedure described in detail in Ref.^14^ and briefly summarized in the following.

The function $D = D\left( t \right)$ is determined by minimizing, point by point along the whole integration interval, the difference between the calculated concentration $r$ (Eqs. 1–6) and the corresponding experimental value (we imposed that such a difference keeps lower than 1%). The result of this procedure is plotted in Fig. 2b, using a 5-day moving average filter.

We extracted the functional form of *D* and all the other relevant model parameters from a fit to the data of hospitalized cases since they are more reliable than the ones concerning the number of people tested positive for the virus. Indeed, the latters represent only a small fraction of the real corresponding concentration values, since they refer to the cases actually detected through the adopted testing procedure (swabs), restricted to a defined relatively small sample of the entire population. In Fig. 2b, the strong reduction of the diffusion coefficient to its minimum value $D_{L} =$ 2.7 × 10^5^ m^2^ day^−1^ is a consequence of the general lockdown in spring 2020, followed by a moderate increase during summer, when the mobility restriction rules were loosened. At the end of September 2020, people mobility increased at a higher rate, due to the resumption of school and work activity in more conventional modalities (compared to those based on work and school from home, experienced during the general spring lockdown). The fast increase of the diffusion coefficient in the first half of October 2020 has triggered the start of the second wave of the Covid-19 epidemic in Italy, accompanied by an exponential growth of the number of hospitalized people (Fig. 2a) in the following month.

On October the 25th, 2020 [arrows in Fig. 2b] the Italian Government enacted further mobility restriction rules that induced a new decrease of the diffusion coefficient. These measures were significantly different Region to Region. A few of them, the so-called *red* Regions, experienced mobility restrictions very similar to the ones adopted during spring lockdown. For other Regions the measures were slightly less restrictive (*orange* Regions) up to a situation characterized by the persistence of a relatively high level of mobility with a limited number of restrictions (*yellow* Regions). The inhomogeneous intensity of the new mobility restriction rules reflects on the circumstance that the diffusion coefficient approached, at the beginning of December 2020, a constant value that was about 1.8 times higher than the one reached during the general, homogeneous spring lockdown, $D_{L}$. Continuous line in Fig. 2b is a fit of the *D* values with a set of logistic functions of the following kind^14^:

7 $$D = D_{2} + \frac{{D_{1} - D_{2} }}{{\exp \left( {\frac{{t - t_{0} }}{{\tau_{c} }}} \right) + 1}}$$

where $t_{0}$ is the time around which the diffusion coefficient changes from *D*_1_ to *D*_2_ and $\tau_{c}$ is the characteristic duration of such variation. In order to follow the time-dependence of $D$, the parameters *D*_1_, *D*_2_, $t_{0}$,$\tau_{c}$ where adjusted to their best fit values within four different time intervals: (i) from February the 24th to June the 4th, 2020 (start of the Covid-19 epidemic monitoring in Italy); (ii) from June the 4th to September the 22nd, 2020; (iii) from September the 22nd to October the 25th, 2020; (iv) for times beyond October the 25th, 2020. In particular, the last change of mobility (beyond October the 25th, 2020) was modeled by setting *D*_1_ = 1.4 × 10^6^ m^2^day^−1^, *D*_2_ = 4.8 × 10^5^ m^2^day^−1^ (i.e., $D_{1} = 5.3 D_{L}$ and $D_{2} = 1.8 D_{L}$), $t_{0} = 260$ days from the start of the Covid-19 epidemic monitoring in Italy, and $\tau_{c} = 7.6$ days.

We used the functional form describing the variation of *D* as a function of time for calculating the expected values *r* (hospitalized people) and *m* (total number of fatalities), through Eqs. (1–6), and by adjusting the other model parameters by a fit to the data of Fig. 2a, with the exception of the characteristic times $\tau_{2}$ (healing of hospitalized people) and $\tau_{3}$ (healing of infected, but not hospitalized people) that were set to the values found in the literature ($\tau_{2} =$ 20 days, $\tau_{3} =$ 14 days)^34^.

For all the calculations we also imposed the following initial conditions for Italy: $r_{0} = 127$/A (i.e. the experimental point at $t = 0$), $p_{0} = r_{0} /\left( {1 - f} \right)$, $c_{0} = p_{0}$, $g_{0} = 0, m_{0} = 0$. Here $A$ indicates the surface of the geographical area. From the best-fit we obtained $f$, $q$, $\tau_{1}$.

The results of such a procedure are the continuous lines plotted in Fig. 2a. The agreement of the theoretical curves with the experimental data is excellent. The best-fit values found for *f* (fraction of the new infected persons that require hospitalization), $\tau_{1}$ (characteristic time for death), *q* (fraction of hospitalized people that die in the characteristic time $\tau_{1}$) were: *f* = 0.35%, $\tau_{1} =$ 7.2 days, *q* = 14% in the time interval February 24th $\le t \le$ May the 13rd, *q* = 10% in the time interval May 13rd $< t \le$ November the 14th, *q* = 15% for $t >$ November the 14th. In the same plots, dashed lines simulated what would have occurred if the diffusion coefficient, after October the 20th, had approached the same value experienced in the occasion of the spring general lockdown.

**Figure 3:**
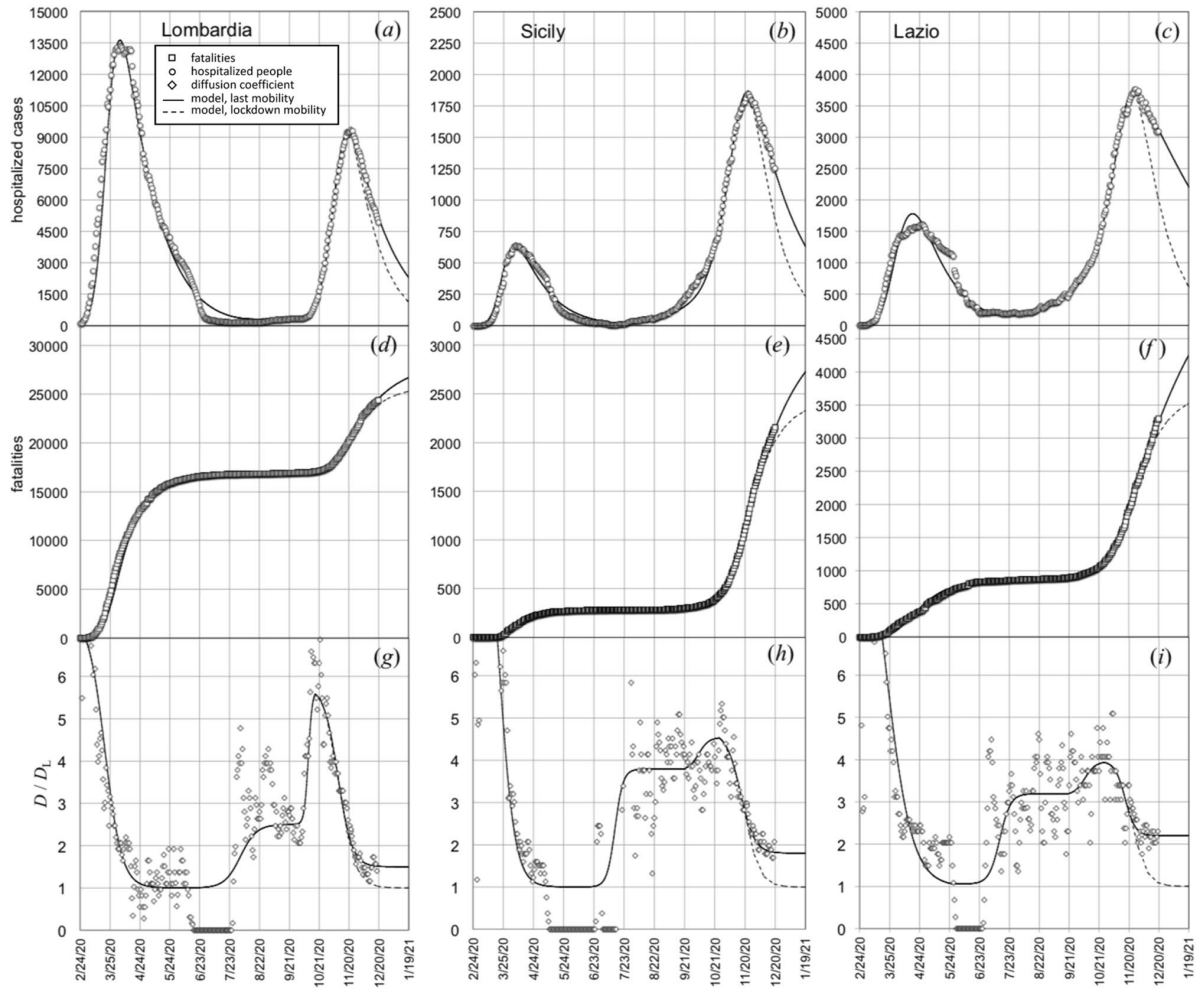
(**a**–**c**) Hospitalized cases before December the 20th, 2020, in Lombardia, Sicilia, and Lazio, respectively. (**d**–**f**) Corresponding number of fatalities. (**g**–**i**) Values of the diffusion coefficient normalized to the ones reached during the first lockdown in spring 2020, for Lombardia, Sicily, and Lazio, respectively. Continuous lines are fit to the data by the present model. Dashed lines simulated of the behaviour we would have observed if the diffusion coefficient had decreased to *D*_L_ (the spring lockdown value) after October the 20th, 2020.

The same analytical procedure was applied to model the evolution of epidemic data in three Italian Regions that, on November the 5th, 2020, were subjected to different mobility restrictions: Lombardia (“red zone”: severe mobility restrictions), Sicilia (“orange zone": medium level of mobility restrictions), Lazio (“yellow zone”: soft mobility restrictions). The results are shown in Fig. 3. The model fits the hospitalized cases [full lines in Fig. 3a–c] and number of fatalities [full lines in Fig. 3d–f] by using the functional time dependences of *D* plotted as continuous lines in Fig. 3g–i. Dashed lines simulated the behaviour we would have observed if the diffusion coefficient had decreased, after October the 20th, 2020, to the corresponding spring lockdown values *D*_L_. We notice that, in the range October the 20th–December the 20th, the diffusion coefficient decreases to a plateau level that is different for the three examined Regions. Compared to the corresponding spring lockdown values *D*_L_, the ratio $D/D_{{\text{L}}}$ approaches 1.5 for Lombardia, 1.8 for Sicily, and 2.2 for Lazio, respectively. Thus, the model allows us to relate the temporal evolution of the epidemic data to the change of people mobility (diffusion coefficient) caused by different levels of restriction severity.

The model fitting parameters are listed in Table 1. Also in this case (as for Italy as whole) we need to use different values of the parameter *q* for the three time intervals (the first range is centered on the first wave of the outbreak, the second one corresponds to the summer characterized by a relatively small number of cases, and the third interval is around the peak of the second wave).

**Table 1:** Parameter values used to fit the theoretical model to the data shown in Fig. 3.

	*f*	τ_1_ (days)	*q*		
Lombardia	0.32%	5.0	16% Feb. the 24th $\le$ *t* < Apr. the 18th	6.5% Apr. the 18th $\le$ *t* < Nov. the 12th	9% *t* $\ge$ Nov. the 12th
Sicily	0.30%	5.1	6% Feb. the 24th $\le$ *t* < Apr. the 18th	2.5% Apr. the 18th $\le$ *t* < Oct. the 25th	11% *t* $\ge$ Oct. the 25th
Lazio	0.28%	6.8	6% Feb. the 24th $\le$ *t* < May the 28th	2.5% May the 28th $\le$ *t* < Oct. the 22th	8.6% *t* $\ge$ Oct. the 22th

### Effect of mobility increase occurred during 2020 holiday season

**Figure 4:**
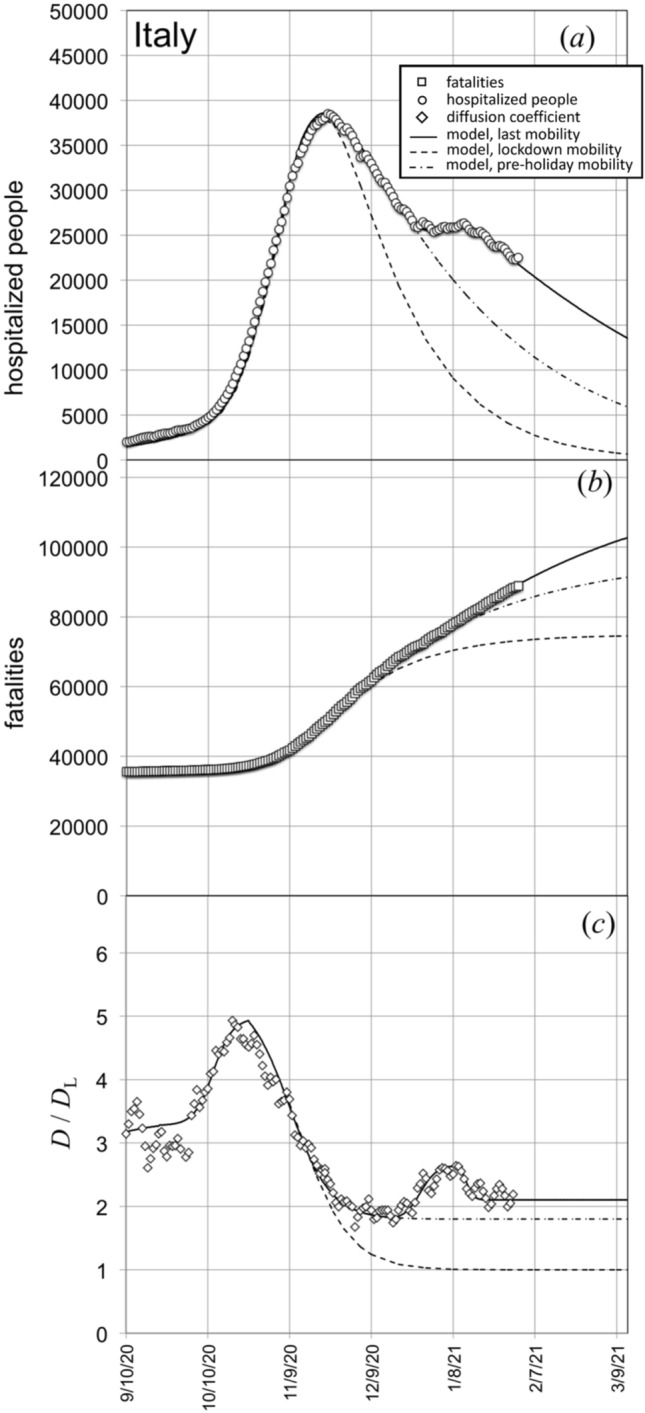
(**a**) Hospitalized people and (**b**) fatalities in Italy in the time range centered on the second wave of the outbreak and on holiday season 2020. (**c**) Evolution of the diffusion coefficient, in the same time range, normalized to the spring lockdown value *D*_L_. Continuous lines fit the present model to the data. Dashed lines correspond to the hypothesis of a general lockdown on October the 20th, 2020. Dot-dashed lines describe the situation we would have experienced if restriction mobility measures had maintained unchanged during holiday season.

After December the 20th, 2020, the Italian Government decided to relax the mobility restriction measures in the occasion of holiday season. The corresponding increase of people mobility reflected in a significant slowdown of the decreasing rate of the hospitalized cases as shown in Fig. 4a.

The proposed model interprets this effect in terms of variation of the diffusion coefficient, with respect to the spring lockdown value *D*_L_. In particular, Fig. 4c indicates that the diffusion coefficient (and the related quantity $\rho_{0} D$, i.e. “average number of people encountered by each person in a day”) increased, during holiday season, from 1.8 to 2.6 times *D*_L_. The reintroduction of more severe mobility restrictions on January the 7th caused a new decrease of *D*. However, these new actions (same in all the National territory) were significantly softer than the ones adopted before December the 20th. This circumstance reflects on the observation that the ratio *D*/*D*_L_ approached, after the holiday season peak, to a value, for Italy as a whole, of about 2.1, higher than the one observed in the pre-peak time interval (*D*/*D*_L_ = 1.8). The corresponding fits of the model to the hospitalized and fatalities cases are plotted as continuous lines in Fig. 4a and b.

The simulation of the scenario for a decrease of the diffusion coefficient, after October the 20th, 2020, to *D*_L_ is also plotted in Fig. 4 (dashed lines), whilst dot-dashed lines simulate the scenario corresponding to a diffusion coefficient constant to the value experienced before holiday season.

**Figure 5:**
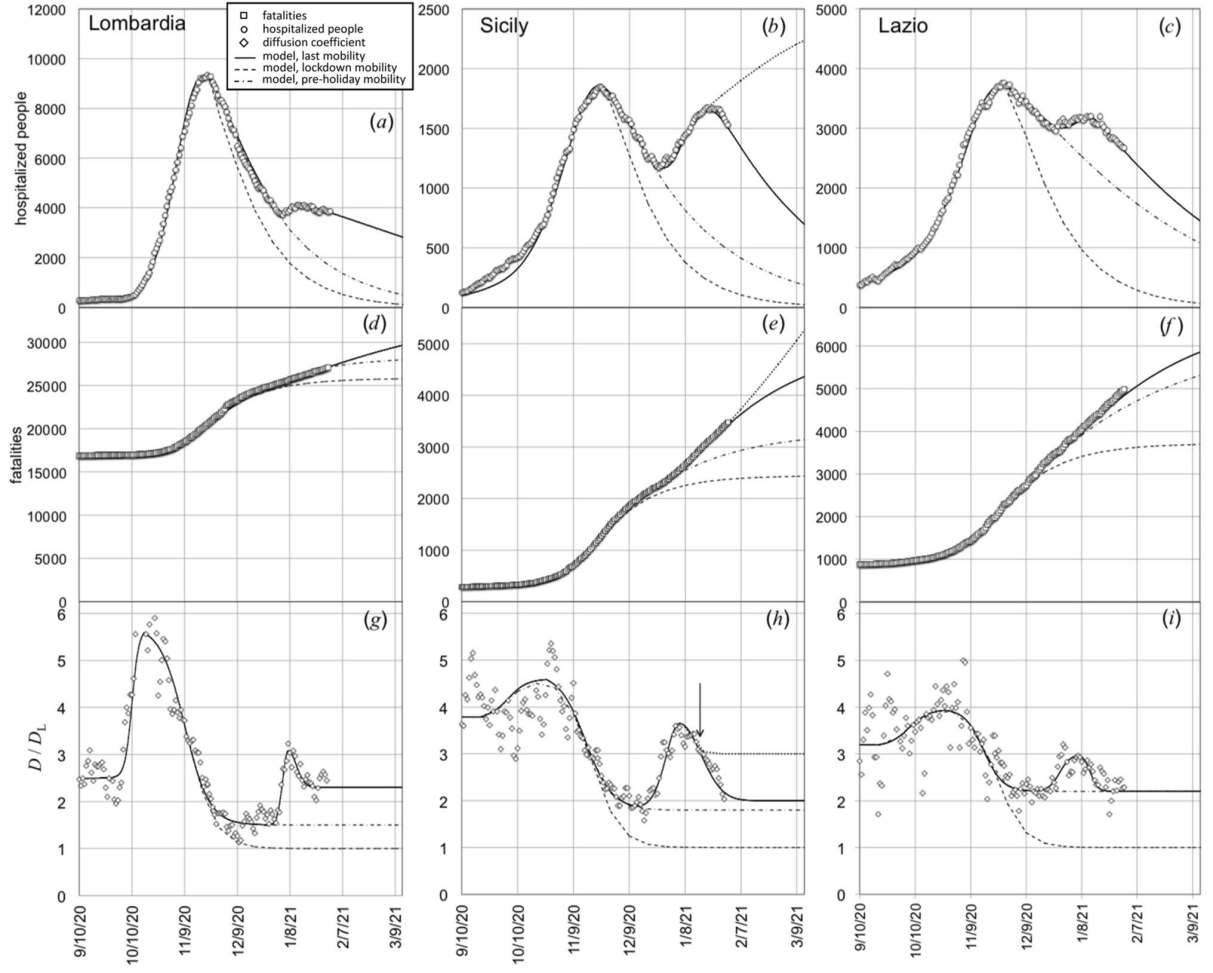
(**a**–**c**) Hospitalized cases in the time range that includes holyday season 2020, in Lombardia, Sicily, and Lazio, respectively. (**d**–**f**) Corresponding number of fatalities. (**g**–**i**) Values of the diffusion coefficient normalized to *D*_L_ Easing of restrictions during holiday season increased the diffusion coefficient, with peaks centered on January the 5th, 2021. Continuous lines fit the present model to the data. Dashed lines correspond to the decrease of *D*, after October the 20th, 2020, to *D*_L_. Dot-dashed lines describe the situation we would have expected if restrictions had maintained unchanged during holiday season. For Sicily, the simulation of a post-peak holiday season diffusion coefficient that decreases to a level as large as 3 times than *D*_L_ is shown (dotted lines).

The variation of *D*/*D*_L_ around the peak of holiday season has not been the same among the different Italian Regions. This is shown in Fig. 5 for Lombardia, Sicily, and Lazio. We notice that only Lazio has returned to a situation with a diffusion coefficient of the pre-holiday season value. Conversely, the ratio *D*/*D*_L_ in Lombardia approached the value of 2.3, significantly larger than the pre-holiday season value (*D*/*D*_L_ = 1.5). Sicily has experienced the most critical situation, with *D*/*D*_L_ that has reached, during holiday season, a peak value as large as 3.6, thus triggering the start of a third wave of the outbreak, well visible in the plot of hospitalized cases shown in Fig. 5b. Indeed, beyond the holiday season peak, the ratio *D*/*D*_L_ for Sicily has exhibited a relatively slow decreasing rate and, on January the 15th [see the arrow in Fig. 5h], it was still at a level of about 3.3. In particular, dotted lines in Fig. 5b, e, h show the results of our simulation under the hypothesis that *D*/*D*_L_ for Sicily had approached, after the holiday season peak, a constant value equal to 3. With this assumption a new wave of the virus epidemic in the Region would occur. On January the 15th, however, the Italian Government imposed for Sicily more severe mobility restrictions (“red zone”), inducing a further decrease of *D/D*_L_ and, as a consequence, the number of hospitalized people decreased as well (Fig. 5b).

In this first part of the discussion, we have presented the capability of the model to evaluate *D* and to compare its value to the different restriction levels in Italy: before, during and immediately after lockdown, during and after summer, during and after holidays. In brief, we have related all the restriction levels to corresponding *D*-values in a quite direct relation. This gives us, as shown in Figs. 2, 3, 4, 5, the possibility of forecast the dynamics of the disease holding the restrictions or modifying them. Some SIR-based models simulated the lockdown dynamics by introducing a quarantine compartment. Lemos-Paiao et al. used this strategy for Portugal^21^ while Lopez and Rodò for Italy and Spain^23^. Note that, in these cases, the outbreak spreading is limited to the first wave of the disease.

There are some other few attempts in literature to describe the time-dependent parameters, in particular the transmission rate related to $\sigma D$, in a time-varying SIR-based model. Cartocci et al.^28^ provided a conceptually similar approach. They adopted a simpler model in terms of positive compartments (considering only positives, not distinguished between hospitalized and non-severe positives) and they obtained quite similar behaviour for the parameter related to restriction, especially in the post lockdown time interval. For the period before and during lockdown, among others, our estimation shows quite reliable results. In fact, we have used the concentration of hospitalized and of fatalities to obtain *D* and *q*. These quantities are more reliable than the concentration of positives, since this last measure depends on many factors (number of swabs, tracking or statistical approach, congestion of the health-care system,…) that can change in each region and in different time periods. During the first outbreak, the few nasal swabs available were mainly used to monitor (elder) severe positives and symptomatics, neglecting mostly asymptomatics.

### Modelling the impact of vaccine immunization

On December the 27th, 2020, Italy started its vaccination campaign. The investigation of the clinic effectiveness of the various vaccines is beyond the aim of this work. Our model, however, can include the influence of vaccination on the time evolution of the virus epidemic by assuming that immunization occurs, in general, about one week later from the inoculation of the second vaccine dose. Then, the concentration $\rho_{i}$ of people immunized by vaccination will increase with time *t* according to the following relationship:

8 $$\rho_{i} \left( t \right) = \mathop \smallint \limits_{{t_{0} + \tau }}^{t} v\left( {t - \tau } \right)dt$$

where *t*_0_ is the immunization time onset, corresponding to the day first person has received the second vaccine dose (January the 16th, 2021, in our case), τ = 7 days is the time interval for getting immunization from the second dose inoculation, and $v\left( {t - \tau } \right)$ is the concentration of people per day that was vaccinated (second dose) on the time corresponding to $t - \tau$.

Under these hypotheses, the influence of vaccine on the time evolution of all the observable variables is taken into account, simply by substituting the term $\left( {\rho_{0} - c} \right)$ with $\left( {\rho_{0} - \rho_{i} - c} \right)$ in Eqs. (1–6). For the daily number of people, $v$, receiving the second vaccine dose, we used the data communicated by the Italian Health Ministry (Ministero della Salute)^32^. In order to simulate the impact of vaccination on the future time evolution of the virus outbreak, the function $v$ was kept constant to the vaccination daily rate experienced the week prior to the date of the last available data, (January the 31st, 2021).

Vaccination is the best weapons we can use to strike virus outbreak and come back to highest levels of mobility in a relatively short time range. In order to investigate how vaccination can help us to increase people mobility, we have simulated several scenarios, shown in Fig. S1, consisting in a progressive increase of the diffusion coefficient to a level as high as the one reached at the end of summer 2020 (*D*/*D*_L_ = 3.25).

These scenarios differ for the used time delays to increase the diffusion coefficient to *D*/*D*_L_ = 3.25. Starting from January the 31st, 2021 such delay is set to (see Fig. S1): 1 month (dotted line), 2 months (dashed line), and 3 months (dot-dashed line). Continuous line refers to a diffusion coefficient *D* = 2.1 *D*_L_.

**Figure 6:**
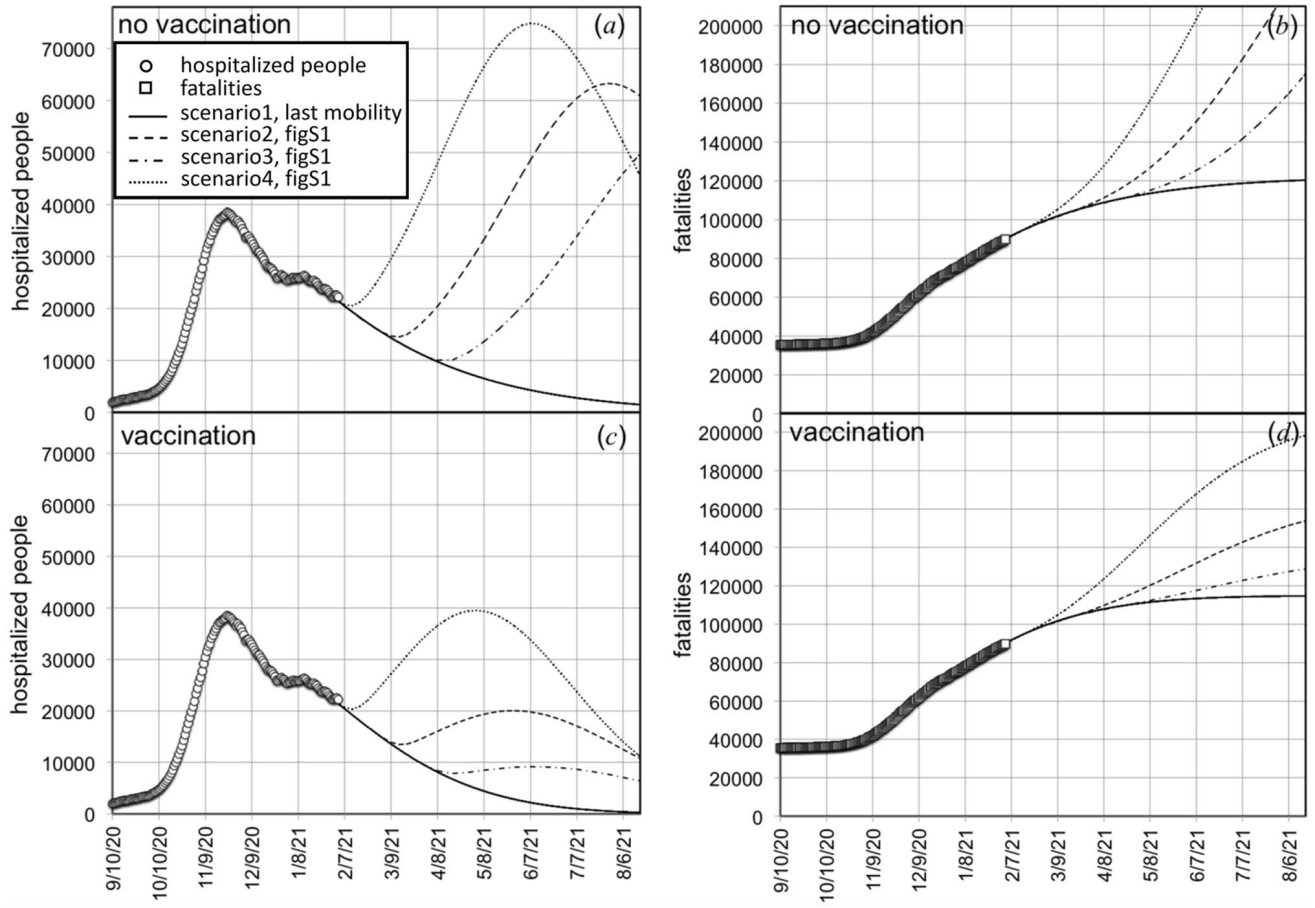
Simulation of the number of hospitalized people and of fatalities due to the increase of people mobility at the level experienced at the end of summer 2020, according to the time evolution of the diffusion coefficient shown in Fig. S1. Calculations in (**a**) and (**b**) were performed in the absence of vaccination, whilst in (**c**) and (**d**) we the effect of vaccine immunization is included assuming that the average vaccination daily rate keeps constant to the value of the last week, prior to January the 31st, 2021.

The simulated number of hospitalized people and of fatalities are plotted in Fig. 6, in the absence (Fig. 6a, b) or in the presence of vaccination (Fig. 6c, d). Maintaining constant the diffusion coefficient to the present low level (*D*/*D*_L_ = 2.1), the vaccine immunization produces only small effects on the decreasing rate of hospitalized people and fatalities, since these values are already decreased by the low mobility. Vaccination beneficial effect is evident when the mobility is higher. It strongly mitigates the amplitude of the third wave peak of the outbreak triggered by the increase of people mobility to the levels measured at the end of summer 2020 (*D*/*D*_L_ = 3.25).

### Reproduction number calculation

The model provides also a self-consistent method to evaluate the reproduction number *R*_T_, i.e. the number of primary infections produced by a single infected person during the time interval she/he remains still positive (we actually assume that a positive human transmits the infection with a constant probability, independently of the symptoms severity). We consider the concentration $n$ of people infected by a positive concentration “probe” $\overline{p}\left( \theta \right),$ initially equal to $\overline{p}_{t}$. The new positives for the virus at the time instant $t$, normalized to $\overline{p}_{t}$ itself, increases, at a time instant $\theta \ge t$, by a quantity $dn$ given by:

9 $$\frac{dn}{{\overline{p}_{t} d\theta }} = \rho_{0} D\sigma \left[ {\rho_{0} - \rho_{i} \left( \theta \right) - c\left( \theta \right)} \right]\frac{{\overline{p}\left( \theta \right)}}{{\overline{p}_{t} }}$$

The function $c\left( \theta \right)$ is determined, for assigned forms of $\rho_{i} \left( \theta \right)$ and $D\left( \theta \right)$, by solving Eqs. (1–6), whilst $P\left( \theta \right) = \overline{p}\left( \theta \right)/\overline{p}_{t}$ decays with time according to the rate equations that describe the process of healing or death of people positives for the virus, i.e.:

10 $$\frac{dP}{{d\theta }} = - \frac{dG}{{d\theta }} - \frac{dM}{{d\theta }}$$

11 $$\frac{dR}{{d\theta }} = - \left( {\frac{q}{{\tau_{1} }} + \frac{1 - q}{{\tau_{2} }}} \right)R\left( \theta \right)$$

12 $$\frac{dM}{{d\theta }} = \frac{q}{{\tau_{1} }}R\left( \theta \right)$$

13 $$\frac{dS}{{d\theta }} = - \frac{S\left( \theta \right)}{{\tau_{3} }}$$

14 $$\frac{dG}{{d\theta }} = \frac{S\left( \theta \right)}{{\tau_{3} }} + \frac{{\left( {1 - q} \right)R\left( \theta \right)}}{{\tau_{2} }}$$

The analytical solution of this system of differential equations is:

15 $$P\left( \theta \right) = \left( {1 - f} \right)\exp \left( { - \frac{\theta }{{\tau_{3} }}} \right) + f\exp \left[ { - \frac{{\tau_{1} + q\left( {\tau_{2} - \tau_{1} } \right)}}{{\tau_{1} \tau_{2} }}\theta } \right]$$

The function $P\left( \theta \right)$, calculated by setting $f$, $q$, $\tau_{1}$, $\tau_{2}$, $\tau_{3}$ to the values used to fit the model to the data of hospitalized people and fatalities for Italy as a whole, is plotted in Fig. S2. $P\left( \theta \right)$ is just but the probability that a single infected individual, contracting the viral infection at a given instant $\theta = 0$, is still positive and able, in turn, to infect susceptible people at a subsequent time $\theta > 0$.

$R_{T}$ is defined as the number of people infected by a single individual, positive for the virus at a certain instant $t$, throughout her/his full lifetime (until healing or death), and then:

16 $$R_{T} = \mathop \smallint \limits_{t}^{ + \infty } \frac{dn}{{\overline{p}_{t} d\theta }}d\theta = \rho_{0} \sigma \mathop \smallint \limits_{t}^{ + \infty } D\left( \theta \right)\left[ {\rho_{0} - \rho_{i} \left( \theta \right) - c\left( \theta \right)} \right]P\left( \theta \right)d\theta$$

Since $R_{T}$ is the result of a time integration, the instant, *T*, at which the determination of *R*_T_ should be referred to is equal to the time average weighted on $dn$, i.e. the number of people that a single positive infects per unit of time throughout her/his lifetime:

17 $$T = \frac{{\mathop \smallint \nolimits_{t}^{ + \infty } \theta \frac{dn}{{d\theta }}d\theta }}{{\mathop \smallint \nolimits_{t}^{ + \infty } \frac{dn}{{d\theta }}d\theta }} = \frac{{\mathop \smallint \nolimits_{t}^{ + \infty } \theta D\left( \theta \right)\left[ {\rho_{0} - \rho_{i} \left( \theta \right) - c\left( \theta \right)} \right]P\left( \theta \right)d\theta }}{{\mathop \smallint \nolimits_{t}^{ + \infty } D\left( \theta \right)\left[ {\rho_{0} - \rho_{i} \left( \theta \right) - c\left( \theta \right)} \right]P\left( \theta \right)d\theta }}$$

**Figure 7:**
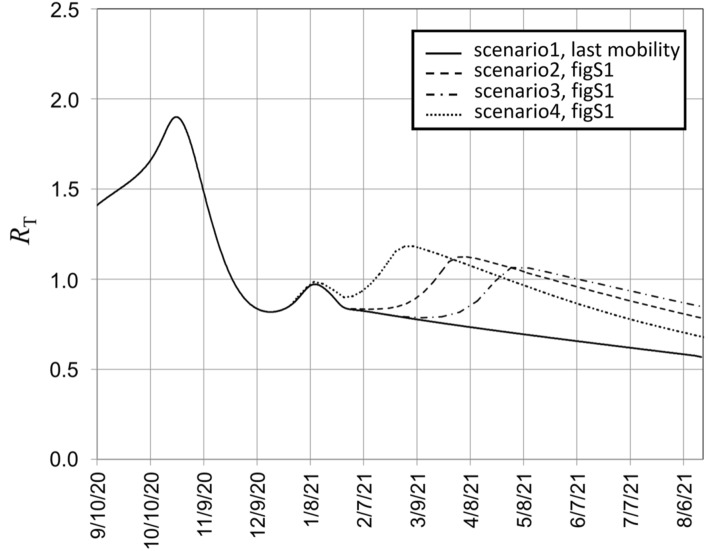
Reproduction number *R*_T_ as a function of time corresponding to the different scenarios of variation of the diffusion coefficient illustrated in Fig. S1.

The time dependence of *R*_T_ calculated by Eq. (16), as a function of the corresponding time *T* expressed by Eq. (17), is plotted in Fig. 7 for the different scenarios of people mobility variations described in Fig. S1. By comparison of Fig. 7 to Figs. S1 and 6, we notice that the increase of the diffusion coefficient to the level experienced at the end of summer 2020 in Italy, causes a reproduction number above 1, responsible then for the triggering of a new wave of the virus outbreak.

We finally observe that the integral term in Eq. (16) can be thought as the average number ${\Gamma }$ of people, not yet infected (“susceptible people”), that a single positive individual meets throughout his lifetime, since the onset of his infection. Thus, we can conclude that *R*_T_ is proportional to ${\Gamma }$, the proportionality constant being $\rho_{0} \sigma$, i.e. the probability of a single infection event.

**Figure 8:**
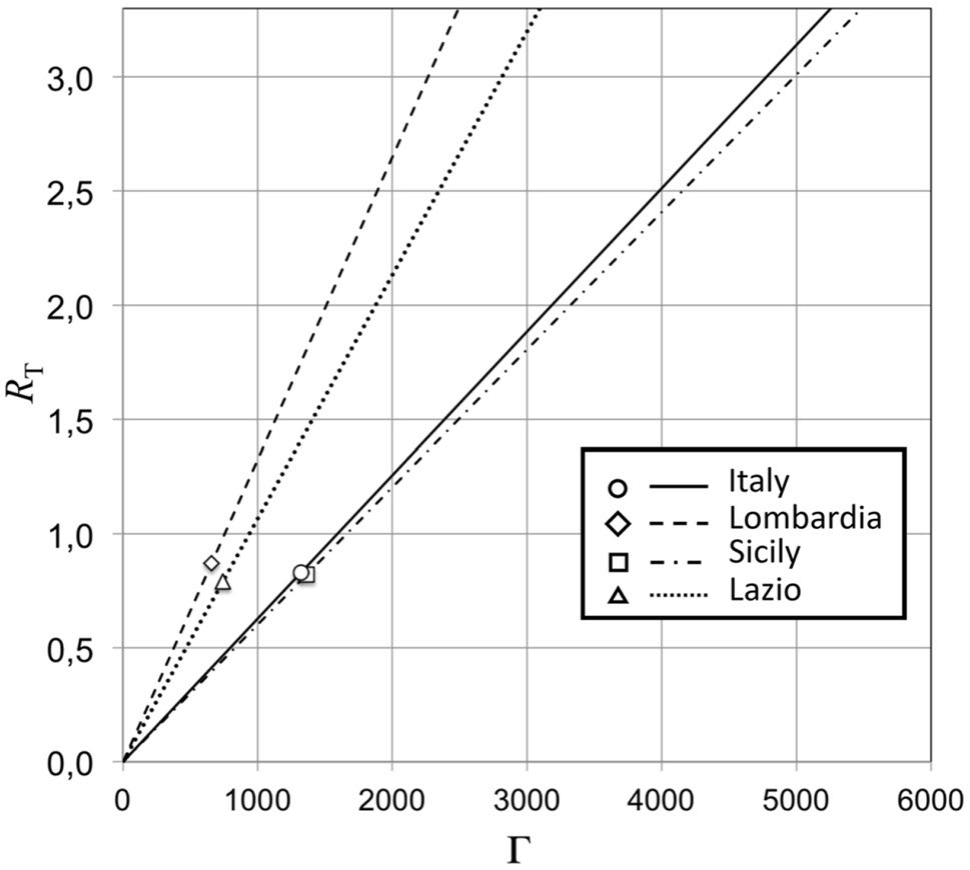
Reproduction number *R*_T_ versus average number Γ of susceptible people that a single individual, positive for the virus, meets throughout her/his lifetime, for Italy (continuous line), Lombardia (dashed line), Sicily (dot-dashed line), and Lazio (dotted line). The present (*R*_T_, Γ) values, as of January the 31th, 2021, are plotted as open circle, open lozenges, open square, and open triangle, respectively.

Nowadays, the Covid-19 outbreak in the Italian Regions we have analyzed (Lombardia, Sicily, and Lazio) is actually characterized by similar values of *R*_T_, all of them being below 1: 0.87 for Lombardia, 0.82 for Sicily, 0.79 for Lazio, 0.83 for Italy as a whole. However, people mobility levels corresponding to these similar *R*_T_ values differ Region by Region. This situation is clearly illustrated in Fig. 8, where *R*_T_ is plotted as a function of Γ (the number of susceptible people that a single positive meets on average throughout his lifetime) for Italy (continuous line), Lombardia (dashed line), Sicily (dot-dashed line), and Lazio (dotted line). The present (*R*_T_, Γ values, as of January the 31th, 2021, are plotted as open circle, open lozenges, open square, and open triangle, respectively. We notice that a same value of *R*_T_ corresponds, for the examined Regions, to different average number of susceptible people met by a single person positive for the virus during his lifetime. Setting people mobility to the very same level experienced nowadays on average in Italy (Γ ~ 1300), will produce in Lombardia a significant increase of *R*_T_ from 0.87 to 1.75, as a direct consequence of the circumstance that the density of inhabitants of Lombardia is double of that of Italy as a whole.

### Effect of virus variants

Our theoretical description can effectively provide simulation of the influence of virus variants on the time evolution of hospitalized cases and fatalities. A virus variant is expected to be more infective and/or or more severe in terms of the fraction of infected people requiring hospitalization. In the former case we should simply increase the value of the infection cross-section $\sigma$, in the latter case it is *f* that has to be changed. In particular, the effect of virus variants can be simulated by assuming that at, a certain date, the new form of the virus, characterized by different values of $\sigma$ and/or *f*, is present in a small fraction of the active positive population. After that time, the concentration $p^{\prime }$ of people positive for the virus variant will vary with time according to the same set of equations, Eqs. (1–6), that are simultaneously used to follow the time variation of the concentration $p$ of people positive for the standard version of the virus. Of course, Eq. (6) has to be modified to take into account for the presence of $p$ and $p^{\prime }$ in the population of infected people. For virus variant with different transmissibility $\sigma^{\prime }$ and $\sigma$, Eq. (6) transforms in:

18 $$\frac{dc}{{dt}} = \rho_{0} D\sigma \left( {\rho_{0} - \rho_{i} - c} \right)p + \rho_{0} D\sigma^{\prime } \left( {\rho_{0} - \rho_{i} - c} \right)p^{\prime }$$

whilst, for virus variant characterized by more severe symptoms but same transmissibility, Eq. (6) becomes:

19 $$\frac{dc}{{dt}} = \rho_{0} D\sigma \left( {\rho_{0} - \rho_{i} - c} \right)\left( {p + p^{\prime } } \right)$$

since the variation $f \to f^{\prime }$ is included in Eq. (2) describing the time evolution of fraction of $p^{\prime }$ that requires hospitalization. In all these approximations, we assume that all the other parameters (*q*, τ_1_, τ_1_, and τ_1_) remain unchanged and that vaccine immunization is still effective for either standard or variant form of the virus.

**Figure 9:**
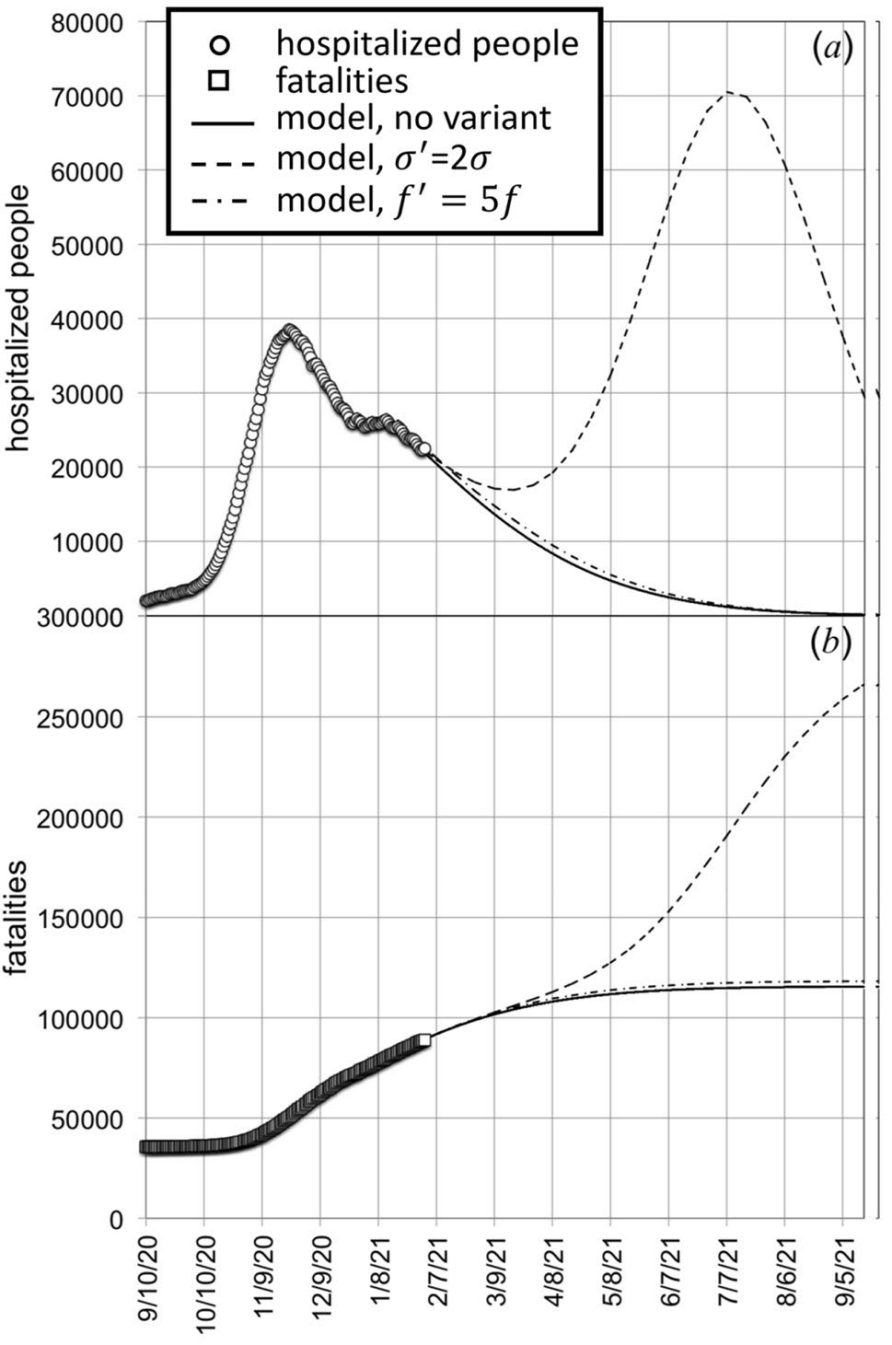
Simulation of impact of virus variant on the time evolution of hospitalized cases (**a**) and fatalities (**b**) in Italy. Calculations were performed by assuming that 1% of the active positives on January the 15th, 2021 were affected by a virus variant characterized by an infection cross-section higher by a factor 2 (dashed lines), or by a virus variant producing more severe symptoms, described by an increase by a factor 5 of the fraction of positives requiring hospitalization (dot-dashed lines). Continuous line is the predicted time evolution of cases in the absence of virus variants at the present level of mobility (*D/D*_L_ = 2.1).

Figure 9 shows the results assuming the presence, on January the 15th, 2021, of people positives to virus variant at a concentration equal to 1% of the total active circulating positives. We performed these calculations by keeping constant the diffusion coefficient to its present value (*D*/*D*_L_ = 2.1) and by considering the case of a virus variant with higher transmissibility ($\sigma^{\prime } = 2\sigma$, dashed line in Fig. 9) or producing more serious symptoms ($f^{\prime } = 5f$, dot-dashed line in Fig. 9). It is evident that the appearance of virus variant having a higher transmissibility is the most dangerous perspective, with respect to the hypothesis that the variant virus characteristics are only limited to the increase of symptoms severity. It should be also emphasized that the increase of cross-section by a factor two is obtained by the increase of the characteristic infection distance, *R*, ($\sigma = \pi R^{2}$), by just 40%.

Since the model is very sensitive to variations of $\sigma D$, by this quantity it is possible to detect the onset of a new variant with a higher transmissibility. Indeed, if $\sigma D$ increases in a time interval in which *D* is kept constant (e.g. when there are no new restrictions or openings) this variation can only be attributed to a higher $\sigma$. Since $\sigma D$ is measured daily, the detection of a new variant is quite immediate. The other methods based on *R*_T_ measurements rely instead on mathematical integrations on several days (Table 2).

**Table 2:** List of symbols used in the present model.

Parameter	Description
$p$	Concentration of people positive for viral infection
$\rho_{0}$	Density of inhabitants
$D$	Surface area covered on average by each person in a day
$\sigma$	Infection cross-section related to the probability of a single infection event ($\sigma = \pi R^{2}$)
$R$	Average distance within which a healthy person can be infected by a positive one
$c$	Total concentration of those who have contracted the virus at the time $t$
$g$	Concentration of healed people (infected people who are tested negative for the virus after a certain time interval from the infection)
$m$	Concentration of fatalities
$r$	Concentration of hospitalized people
$f$	Fraction of the new positive cases requiring hospital care
$q$	Fraction of hospitalized people dying in a characteristic time $\tau_{1}$
$\tau_{1}$	Characteristic time associated to $q$
$1 - q$	Fraction of hospitalized people healing in a characteristic time $\tau_{2}$
$\tau_{2}$	Characteristic time associated to $1 - q$
$s$	Concentration of people not exhibiting serious symptoms (not requiring hospital care) until complete healing in a characteristic time $\tau_{3}$
$\tau_{3}$	Characteristic time associated to $s$
$\rho_{i}$	Concentration of people immunized by vaccination
*t*_0_	Immunization time onset, corresponding to the day first person has received the second vaccine dose
τ = 7 days	Time interval for getting immunization from the second dose inoculation
$v\left( {t - \tau } \right)$	Concentration of people per day that was vaccinated (second dose) on the time corresponding to $t - \tau$
$n$	Concentration of people infected by a concentration “probe”$\overline{p}\left( \theta \right)$
$\overline{p}\left( \theta \right)$	Concentration “probe”
$\overline{p}_{t}$	Concentration “probe” at $\theta = 0$
$R_{T}$	Number of people infected by a single individual, positive for the virus at the certain instant $T$, throughout his full lifetime (until healing or death)
$\sigma^{\prime }$	Infection cross-section related to the probability of a single infection event for the virus variant
$p^{\prime }$	Concentration of people positive for viral variant
$f^{\prime }$	Fraction of the new positive cases, related to virus variant, requiring hospital care

Finally, since the model calculates self-consistently $\sigma^{\prime }$, it also describes the incidence of the new variant, i.e. the ratio $p^{\prime } /p$. Moreover, if the experimental data about each variant are separately available, e.g. through genomic sequencing, it is possible to perform a very precise fine-tuning of the value of $\sigma^{\prime }$, of the order of 1‰.

## Conclusions

In conclusion, we have shown that the spread of COVID-19 virus can be successfully described by a compartmental model,based on the assumption that the probability of a single infection event is given by the product between the density of inhabitants and a cross-section measuring the distance within which a person positive for the virus can infect a healthy one. Through the model, it is possible to relate the variation of observed hospitalized cases and fatalities to the modification of the mobility restriction measures, by comparing the present behavior to that already experienced during the first wave of the outbreak. The model includes the effect of vaccine immunization and the role of possible virus variants in propagating the infection. The possibility to simulate the time evolution of the observed cases as a function of a diffusion coefficient function is a powerful tool to investigate the best tradeoff between increasing people mobility and effects of vaccination and/or virus variants in order to keep under control the spread of Covid-19 outbreak.

## Supplementary Information

Supplementary Information.

## Data Availability

Data and code are available at GitHub (https://github.com/anmio/covid_italy). For each figure, the experimental dataset is indicated in the corresponding folder ‘./figNNN’, where NNN is the figure number.
