# Supplementary material for: Simulation of the impact of people mobility, vaccination rate, and virus variants on the evolution of Covid-19 outbreak in Italy
Source: Sci Rep. 2021 Dec 1;11:23225. doi: 10.1038/s41598-021-02546-y (PMC8636642; doi:10.1038/s41598-021-02546-y)
Supplement: Supplementary file 1 — Supplementary Information. [file 41598_2021_2546_MOESM1_ESM.docx]

**Supplementary information for:**

**Simulation of the impact of people mobility, vaccination rate, and virus variants on the evolution of Covid–19 outbreak in Italy**

Corrado Spinella^1^ and Antonio Massimiliano Mio^1,2,*^

^1^ Dipartimento di Scienze Fisiche e Tecnologie per la Materia, Consiglio Nazionale delle Ricerche, Piazzale Aldo Moro 7, I–00185 Rome (Italy)

^2^ Institute for Microelectronics and Microsystems (IMM), Consiglio Nazionale delle

Ricerche (CNR), VIII Strada 5, I-95121 Catania, Italy

[corrado.spinella@cnr.it](mailto:corrado.spinella@cnr.it)

[antonio.mio@cnr.it](mailto:antonio.mio@cnr.it)

**Corresponding Author**

Antonio Massimiliano Mio

Tel.: +39 095 5968 250

e-mail: [antonio.mio@cnr.it](mailto:antonio.mio@cnr.it)


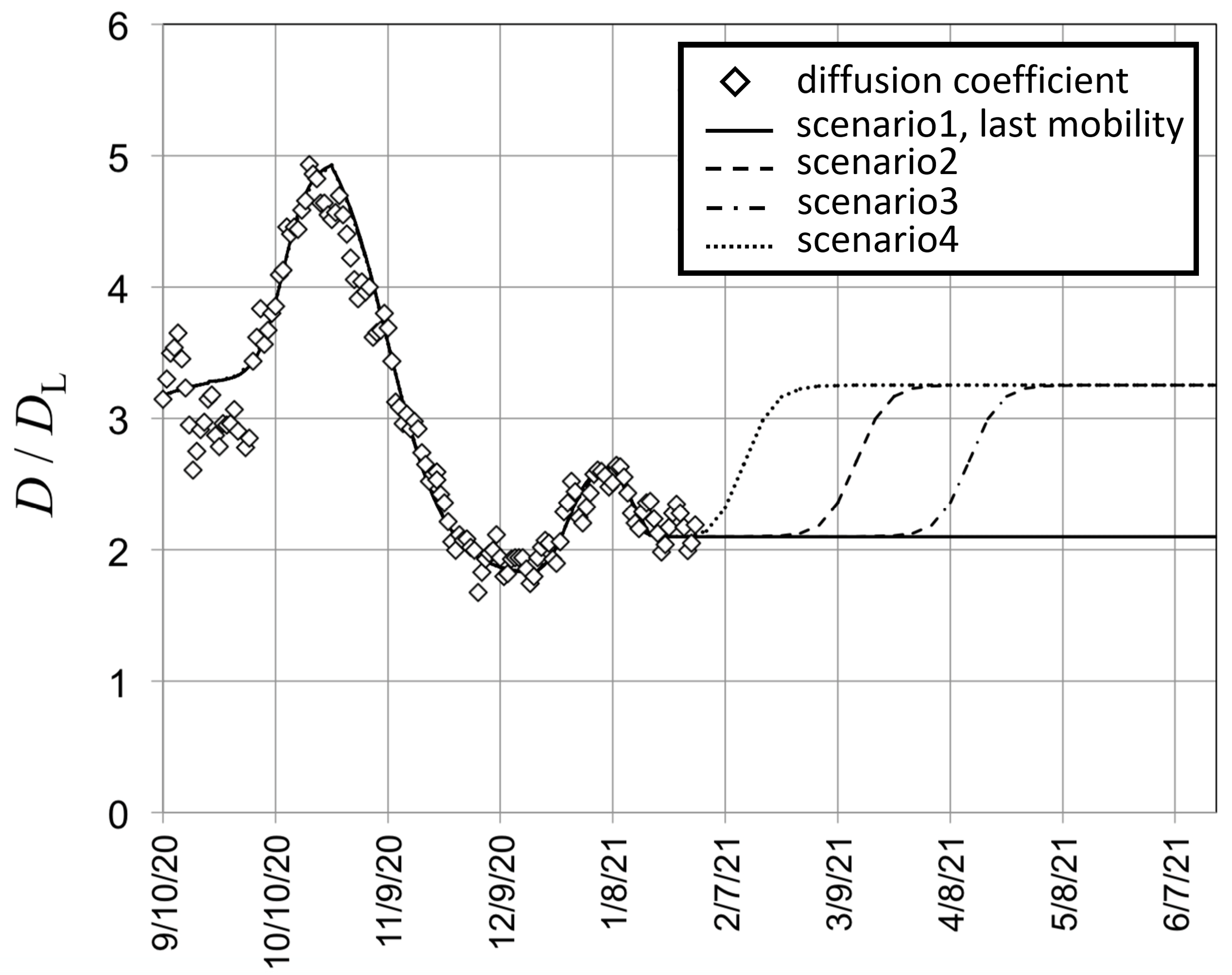


Fig. S1 Diffusion coefficient used for modelling people mobility during the second wave (in course) of the Covid–19 outbreak in Italy. The values are normalized to *D*_L_, i.e. the level reached during the general lockdown of spring 2020. Dotted, dashed, and dot–dashed lines represent different possible scenarios of easing people mobility restrictions to allow an increase of the diffusion coefficient to the level experienced at the end of summer 2020 (*D*/*D*_L_ = 3.25). Continuous line describes the situation according to which the diffusion coefficient is maintained constant to the value of January the 31^th^, 2021 (*D*/*D*_L_ = 2.1)


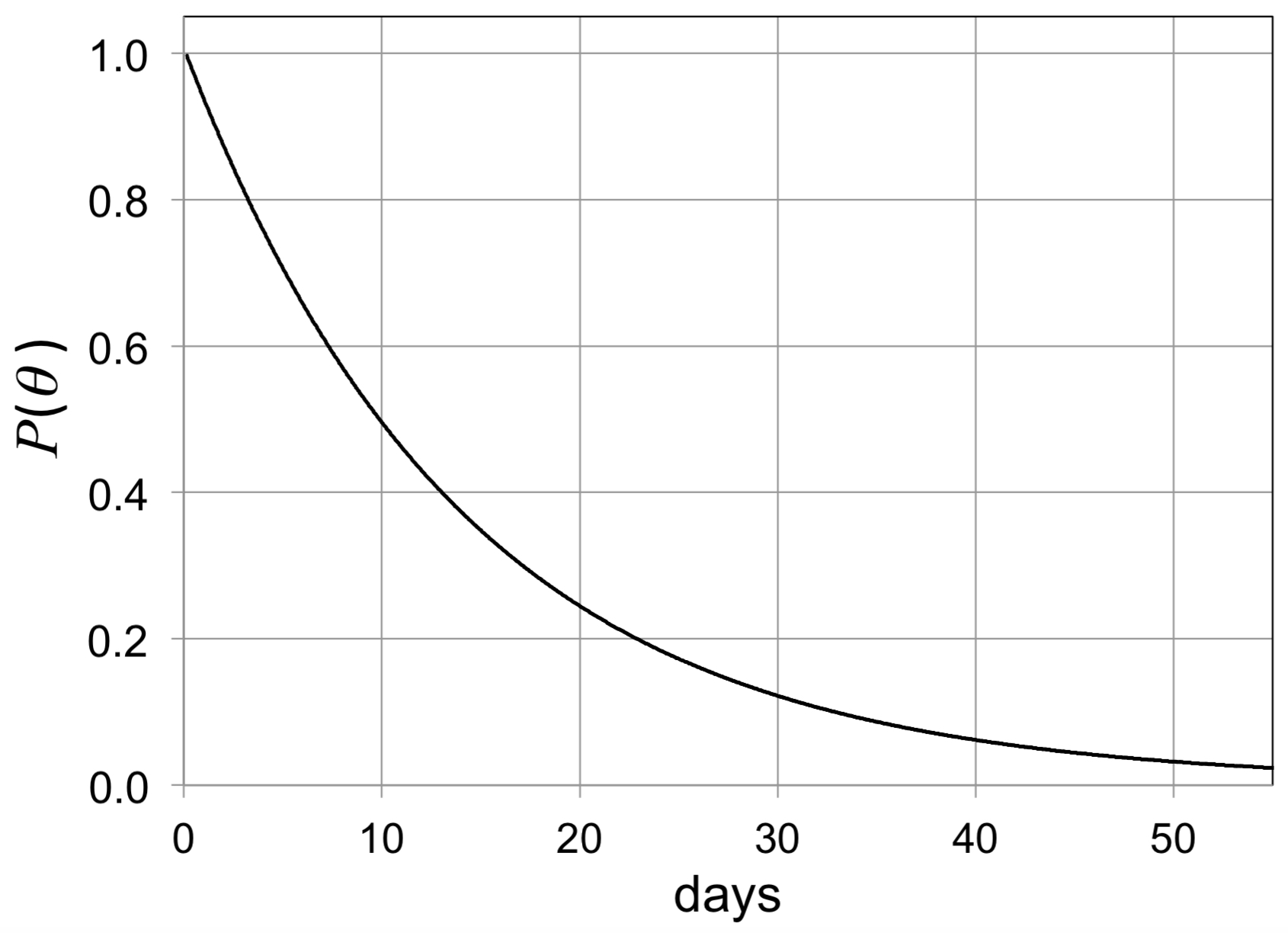


Fig. S2 Calculated probability that a single infected individual is still positive for the virus (and then able to infect, in turn, susceptible people) days after the initial instant of his infection.
